# Urate Oxidase Purification by Salting-in Crystallization: Towards an Alternative to Chromatography

**DOI:** 10.1371/journal.pone.0019013

**Published:** 2011-05-11

**Authors:** Marion Giffard, Natalie Ferté, François Ragot, Mohamed El Hajji, Bertrand Castro, Françoise Bonneté

**Affiliations:** 1 Centre Interdisciplinaire de Nanoscience de Marseille, UPR3118 CNRS, Aix-Marseille Université, Marseille, France; 2 Biotechnology Department, Sanofi-Aventis, Aramon, France; 3 Analytical Sciences Department, Sanofi-Aventis Research and Development, Montpellier, France; Massachusetts Institute of Technology, United States of America

## Abstract

**Background:**

Rasburicase (Fasturtec® or Elitek®, Sanofi-Aventis), the recombinant form of urate oxidase from *Aspergillus flavus*, is a therapeutic enzyme used to prevent or decrease the high levels of uric acid in blood that can occur as a result of chemotherapy. It is produced by Sanofi-Aventis and currently purified via several standard steps of chromatography. This work explores the feasibility of replacing one or more chromatography steps in the downstream process by a crystallization step. It compares the efficacy of two crystallization techniques that have proven successful on pure urate oxidase, testing them on impure urate oxidase solutions.

**Methodology/Principal Findings:**

Here we investigate the possibility of purifying urate oxidase directly by crystallization from the fermentation broth. Based on attractive interaction potentials which are known to drive urate oxidase crystallization, two crystallization routes are compared: a) by increased polymer concentration, which induces a depletion attraction and b) by decreased salt concentration, which induces attractive interactions via a salting-in effect. We observe that adding polymer, a very efficient way to crystallize pure urate oxidase through the depletion effect, is not an efficient way to grow crystals from impure solution. On the other hand, we show that dialysis, which decreases salt concentration through its strong salting-in effect, makes purification of urate oxidase from the fermentation broth possible.

**Conclusions:**

The aim of this study is to compare purification efficacy of two crystallization methods. Our findings show that crystallization of urate oxidase from the fermentation broth provides purity comparable to what can be achieved with one chromatography step. This suggests that, in the case of urate oxidase, crystallization could be implemented not only for polishing or concentration during the last steps of purification, but also as an initial capture step, with minimal changes to the current process.

## Introduction

It is commonly recommended in the field of protein crystallography that a protein solution be purified very thoroughly, in order to maximize chances of successful crystallization. However, crystallization is a technique that has itself long been used in the purification of substances. It has been shown with proteins that crystallization can occur from impure solutions [1], [2], which suggests that crystallization may be an efficient, fast and cost-effective way to purify and concentrate a protein, as is already done with small molecules.

In this paper, we investigate the possibility of using crystallization as a purification step for a therapeutic enzyme, rasburicase, the recombinant urate oxidase from *Aspergillus flavus*. Urate oxidase (uricase, EC 1.7.3.3, uox) is a 135 kDa tetramer with identical subunits having a molecular mass of about 34 kDa. It is responsible for the first step in the degradation of uric acid to allantoin. It is found in a variety of organisms, but its expression is absent in humans and many primates, owing to several specific mutations and deletions [3]. Such mutations may have had an evolutionary function, as uric acid has antioxidant properties that protect the body against neurological degenerative diseases and age-related cancers [4]. Nevertheless, an accumulation of uric acid can lead to gout and, in some extreme cases, to acute hyperuricaemia. Consequently, urate oxidase is used as a protein-based drug [5], Fasturtec® (Sanofi-Aventis), a recombinant urate oxidase (rasburicase) from *Aspergillus flavus* expressed in a genetically modified *Saccharomyces cerevisiae* strain [6]. It is prescribed to prevent renal failure in patients initiating chemotherapy due to rapid tumor lysis or shrinkage.

However, impurities introduced during the preparation of drugs can lead to immunogenicity and hypersensitivity [7]. Using rasburicase to replace pure urate oxidase extracted from *Aspergillus Flavus* has dramatically decreased impurity in the drug and increased its specific activity [6]. To date, urate oxidase is purified using multiple steps of concentration and chromatography [5]. In the biopharmaceutical industry, purity requirements are very stringent and the protein of interest undergoes several chromatographic steps before it can be considered pure. The cell lines used for the upstream process produce a high yield of protein, and the large quantity of protein produced increases the feasibility of crystallization in the downstream process, making it a good candidate as an alternative to one or more of the chromatography steps. As crystallization occurs more easily in pure solutions, it is normally only considered for use as a polishing step at the end of the downstream process. However, from a cost/efficiency point of view, introducing it as a first step to extract and concentrate the protein of interest from the fermentation broth would be advantageous, since it can be performed inexpensively on very large volumes.

Selecting the most appropriate crystallization methods for protein purification is vital. A protein can be considered to be a polyelectrolyte, meaning that a decrease in solubility via a pH shift triggers crystallization and makes purification possible [8], [9]. Adding salt can also be a very effective way to purify a protein by crystallization via the salting-out effect [10]–[14]. It should be noted that the salt used for purification is not necessarily the most effective according to the Hofmeister series, probably because it is chosen in order to decrease selectively the solubility of the protein of interest without decreasing the solubility of the host cell proteins. It has also been shown that the addition of a neutral and non-absorbing polymer such as Poly Ethylene Glycol (PEG) can purify a protein solution via the depletion effect [15]–[17]. Various polymer sizes and concentrations have been used; however in these cases the targeted proteins are usually of high molecular weight (>100 kDa). Finally, a temperature shift could, in principle, also be used as a crystallization method, but to our knowledge there is no example of protein purification where temperature per se triggers the crystallization. However, low temperature plays an important role in preventing the protein denaturation in a purification process where a change of solvent induces crystallization [18].

Knowledge of the solubility of the protein of interest is one of the main requirements in order to assess the use of crystallization as a step in a downstream process. Indeed, a thorough study of solubility is a prerequisite to the successful determination of the crystallization conditions. But it also identifies suitable conditions for crystal dissolution, if this is required in subsequent purification steps. Determining solubility conditions avoids uncontrolled precipitation, liquid-liquid phase separation or denaturation during fermentation or concentration operations. Finally, solubility values determine the highest achievable crystallization yield: for instance, if the protein concentration prior to crystallization is 10 mg/mL and the solubility after crystallization is 5 mg/mL, the yield cannot exceed 50%; if the solubility after crystallization is 1 mg/mL, the yield could theoretically reach 90%. In principle, in both cases, the protein contained in the supernatant could be recycled in the next crystallization batch. This recycling may, however, lead to quality issues. Thus, it would be preferable to have low final solubility and obtain the highest possible yield in a single batch.

The large number of structures available in the Protein Data Bank, which have been determined by crystallography suggests that many proteins can be crystallized. This raises the question of why all the proteins of industrial interest with known crystallization conditions are not purified via crystallization. A possible explanation is that some of these crystallization conditions are not compatible with a downstream process (additives which are not pharmaceutically acceptable even in trace amounts; or which decrease the biological activity of the protein; or which are too expensive to be widely used). In addition, protein crystals generally contain a lot of water (20 to 70%), and may therefore be too fragile to be handled and separated from the supernatant. This water can also contain impurities of lower molecular weight which would decrease the effectiveness of the purification. Finally, protein crystals are often small; they would need to be at least 20 µm in size and to have a homogeneous size distribution in order to ease the process of separation from the supernatant (by filtration or centrifugation).

## Results and Discussion

**Figure 1:**
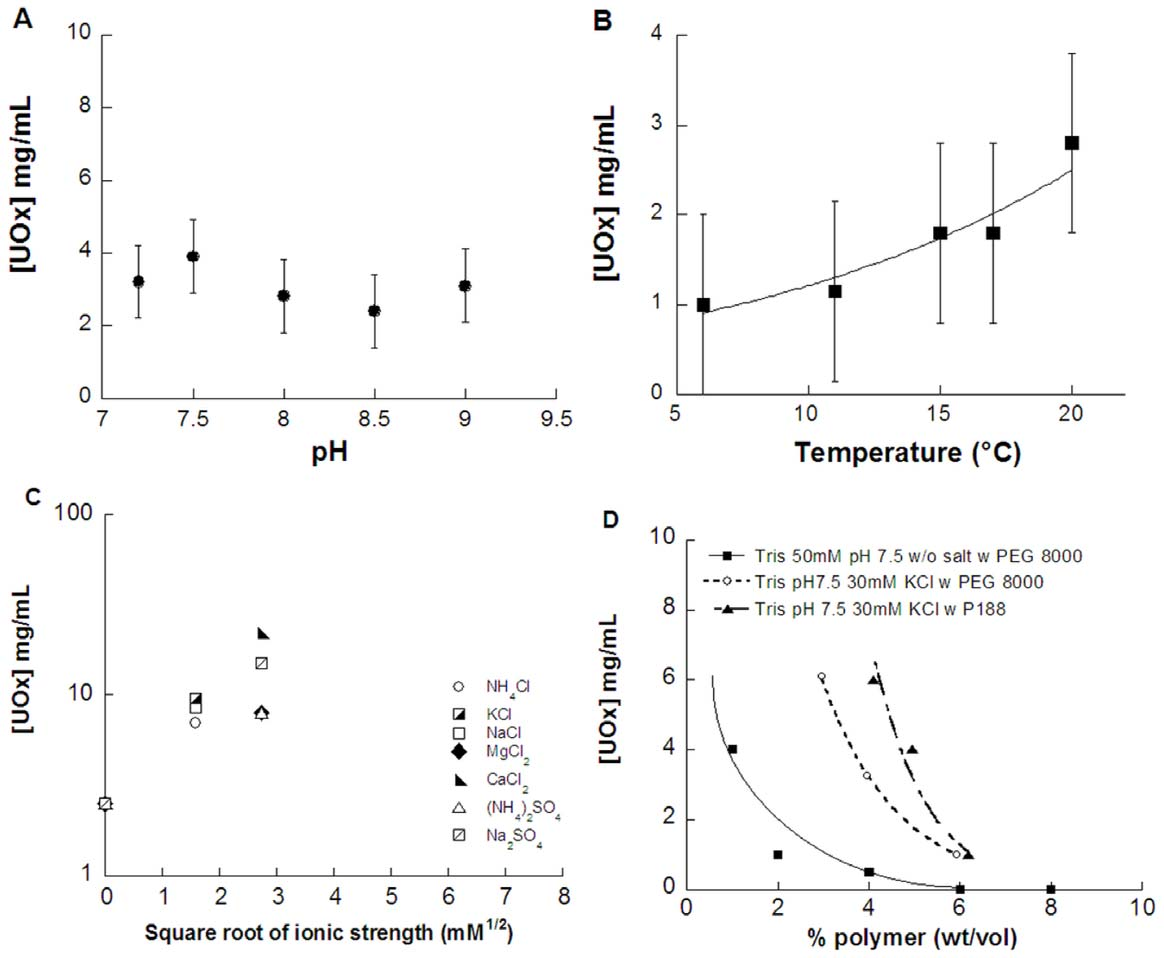
Solubility variations of the recombinant urate oxidase. A) Solubility of urate oxidase as a function of pH in 50 mM Tris buffer at 20°C without added salt. B) Solubility of urate oxidase as a function of temperature in 50 mM Tris buffer pH 8, taking into account variation of pH as a function of temperature with tris buffer (ΔpH/ΔT = −0.03°C^−1^). C) Solubility of urate oxidase as a function of salt type and ionic strength in 50 mM Tris buffer pH 8 at 20°C. D) Solubility of urate oxidase as a function of polymer addition (PEG 8000 or Poloxamer P188) with and without salt at 20°C.

Crystallization of both pure extractive and recombinant urate oxidase has been extensively studied [19]–[22], in particular via thorough interaction potential studies [22]–[25]. It has been shown that attractive interaction potentials between proteins in solution drive the crystallization process [25]–[29]. Although urate oxidase crystallizes under attractive conditions in the presence of polymer, it can also crystallize under attractive conditions in Tris buffer alone, without the addition of any crystallizing agents (salt or polymer) [22]. In this latter case, its solubility is low (3 mg/mL) and does not vary significantly with either temperature or with pH, in the range where the tetrameric form of urate oxidase is stable and active (Figure 1 A, B).

In contrast, the addition of salt in quantities of from a few mM up to at least 1 M dramatically increases urate oxidase solubility (Figure 1 C), which has been described as a strong salting-in effect [22]. The difference is such that the protein is extremely soluble in a 50 mM sodium phosphate buffer of pH 8 (at least 100 mg/mL), whereas it is soluble only up to 3 mg/mL in a 50 mM Tris buffer of pH 8 without any added salt. This difference is due the binding of cations at four binding sites on the surface of the protein, which changes its net charge thereby increasing repulsive interactions and urate oxidase solubility. We have already shown that decreasing salt concentration of a urate oxidase solution via dialysis decreases protein solubility and is one way to trigger crystallization. The crystal shape obtained also depends on the salt used [22].

Another way to decrease urate oxidase solubility and trigger its crystallization is to add common non-adsorbing polymers, such as polyethylene glycols. Non-adsorbing polymers induce a depletion attraction which favors crystallization [19], [23], [24]. We have also recently shown [21] that, in the case of urate oxidase, effective crystallization can be achieved by replacing the polyethylene glycol 8000 by another nonionic polymer, namely poloxamer 188 (Figure 1 D). This amphiphilic polymer, similar in size to PEG 8000, is composed of two poly(oxyethylene) blocks and a central poly(oxypropylene) block with a molecular weight of 8400 g/mol and has a critical micellar concentration (cmc) of about 0.1% w/v [30]. It acts, therefore, at low concentrations (below its cmc) as a solubilizing agent, i.e. inducing repulsive interactions, and at high concentrations (above its cmc) as a crystallizing agent, inducing attractive interactions, without adverse effects on the protein structure or activity [21]. As poloxamer 188 has been used for a long time in the formulation of urate oxidase [31], we chose it over PEG 8000 for crystallization trials.

In comparing these two strategies for crystallizing urate oxidase from impure or partially purified solutions, it was thus vital to respect the following conditions: i) The concentration of urate oxidase prior to crystallization must be high enough, between 10 and 15 mg/mL, and the urate oxidase solubility after crystallization lower than 3 mg/mL in order to reach a yield higher than 70%; ii) Crystals must be sufficiently massive to be separated from the supernatant. We have previously shown that the addition of certain salts to the crystallization medium can induce the growth of such crystals [22]. Since ammonium chloride is already present in the buffer composition of the current urate oxidase purification process by chromatography, it was chosen as a preferred salt; iii) No substrate or inhibitor should be used. For example 8-azaxanthine, a well-known competitive inhibitor [32], could not be used in the crystallization conditions in this process despite the fact that, by stabilizing the active site, it favors crystallization of relatively massive good quality crystals.

**Figure 2:**
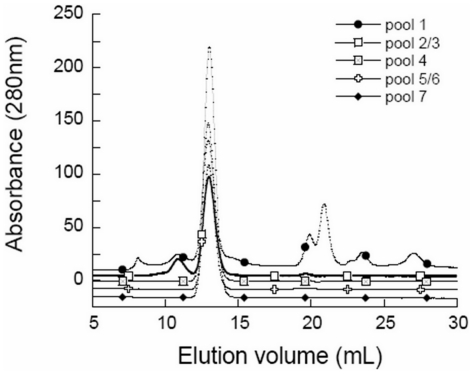
Size exclusion chromatography analysis of the 5 urate oxidase pools from the downstream process. Size exclusion chromatography analysis of the 5 urate oxidase pools from the downstream process. Each pool is analyzed on a Superdex 200 GL column eluted in 50 mM sodium phosphate buffer pH 8 with UV-Vis detection at 280 nm.

**Figure 3:**
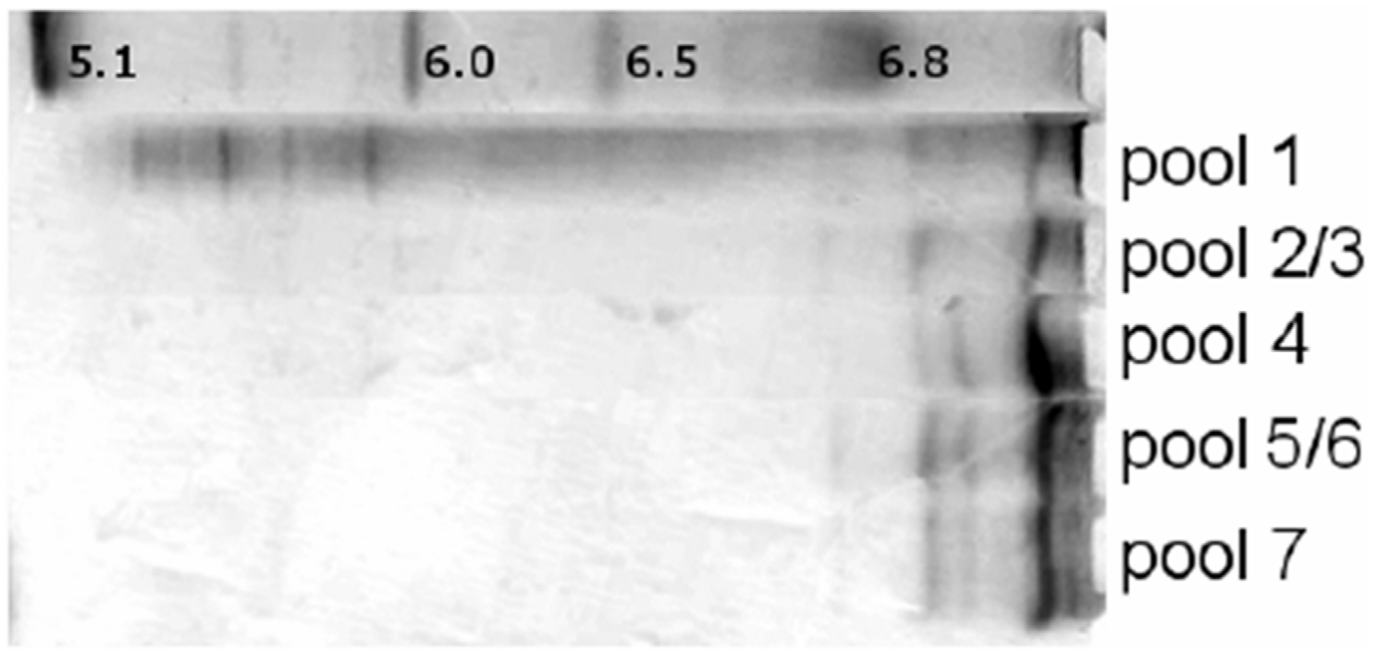
IEF analysis of the 5 urate oxidase pools from the downstream process. IEF analysis of the 5 urate oxidase pools from the downstream process. Pool 2/3 lane identical to that of pool 7, suggesting that the 230 kDa peak corresponds to octomers of urate oxidase.

The urate oxidase was sampled after each step of the downstream process, referred to as pools 1 to 7, pool 1 being the fermentation broth dialyzed and concentrated in Tris buffer prior to the first chromatography step, pool 2 or 3, the solution after the first chromatography step, and pool 7 the purest solution. The different steps of the current process were analyzed by size exclusion chromatography (SEC) (Figure 2), isoelectrofocusing (IEF) (Figure 3) and activity assays. According to the gel filtration column calibration where the active form of urate oxidase is a 135 kDa tetramer, the pool 1 chromatograph presents impurities which are mainly aggregates with sizes higher than 670 kDa, large proteins of roughly 230 kDa, probably urate oxidase octamers (assessed by the activity test) and several unidentified host cell proteins or degradation products of molecular weights lower than 135 kDa. The first chromatography step, which yields pools 2 and 3 in the current process, increases the urate oxidase purity up to 85% (assessed by SEC). The 15% of impurities remaining consist mainly of urate oxidase octamers, as shown by their molecular weight. The second step of chromatography, which yields pool 4, purifies the urate oxidase further up to 99%. The last two chromatography steps are polishing steps.

Despite these two polishing steps, the most purified form of urate oxidase, i.e. pool 7, presents several isoforms (Figure 3) identical to those found in pool 4,5 and 6. The IEF profile of pool 2, which is very similar to the IEF profile of pool 7, also suggests that impurities observed present in this pool are modified forms of urate oxidase. This is in agreement with the hypothesis supported by the SEC data, which shows that pool 2 contains native urate oxidase tetramers and urate oxidase octamers (double MW according to the SEC calibration and similar IEF profile) and no longer contains host cell proteins or aggregates of host cell proteins.

**Figure 4:**
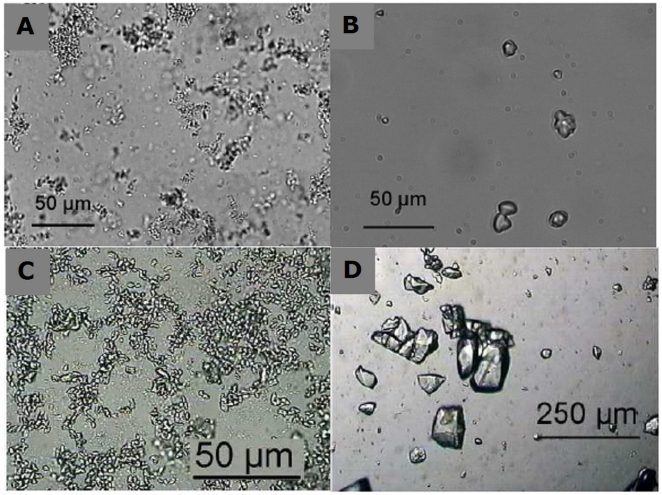
Crystallization trials of urate oxidase from pool 1 and pool 3 either via addition of poloxamer or via reverse dialysis. Crystallization trials of urate oxidase from pool 1 and 3. Top) Crystallization conditions of pool 1. A) c_UOx_≈35 mg/mL, 5% poloxamer 188, NH_4_Cl 20 mM, Tris 5 mM pH 8.5. B) c_UOx_≈40 mg/mL, Tris 5 mM pH 7.5, 20 mM NH_4_Cl vs. Tris 5 mM pH 7.5. Bottom) Crystallization of pool 3. C) c_UOx_≈11 mg/mL, 2.5% poloxamer 188, NH_4_Cl 45 mM, Tris 50 mM pH 7.5. D) c_UOx_≈68 mg/mL 100 mM NH_4_Cl, Tris 50 mM pH 7.5 vs. Tris 50 mM pH 7.5.

**Figure 5:**
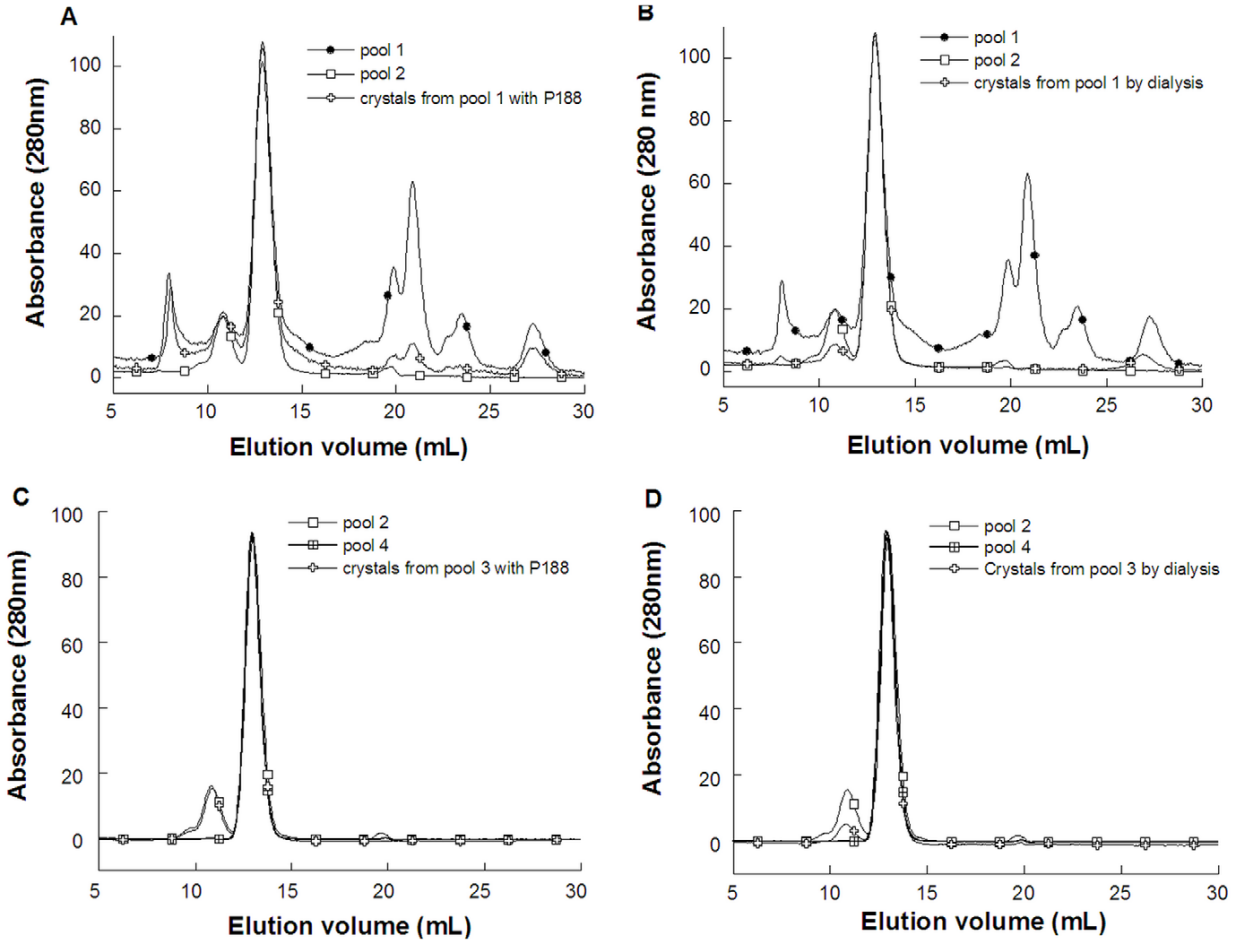
Size exclusion chromatography analysis of urate oxidase crystal content versus chromatography steps 1 and 2. (top) SEC analysis of the crystal content of urate oxidase from pool 1 (unfilled cross) compared to pool 1 (filled circle) and pool 2 (unfilled square): crystallization conditions were (left) 35 mg/mL urate oxidase with 5% poloxamer 188 in 5 mM Tris pH 8.5, 50 mM NH_4_Cl; (right) 40 mg/mL urate oxidase in 5 mM Tris pH 8.5, 50 mM NH_4_Cl, dialyzed against Tris 5 mM pH 8; (bottom) SEC analysis of the crystal content of urate oxidase from pool 3 (unfilled cross) compared to pool 2 (unfilled square) and pool 4 (crossed square): crystallization conditions were (left) 11 mg/mL urate oxidase with 2.5% poloxamer 188 in 5 mM Tris pH 8.5, 50 mM NH_4_Cl; (right) 68 mg/mL urate oxidase in 5 mM Tris pH 8.5, 50 mM NH_4_Cl, dialyzed against 5 mM Tris pH 8.

**Figure 6:**
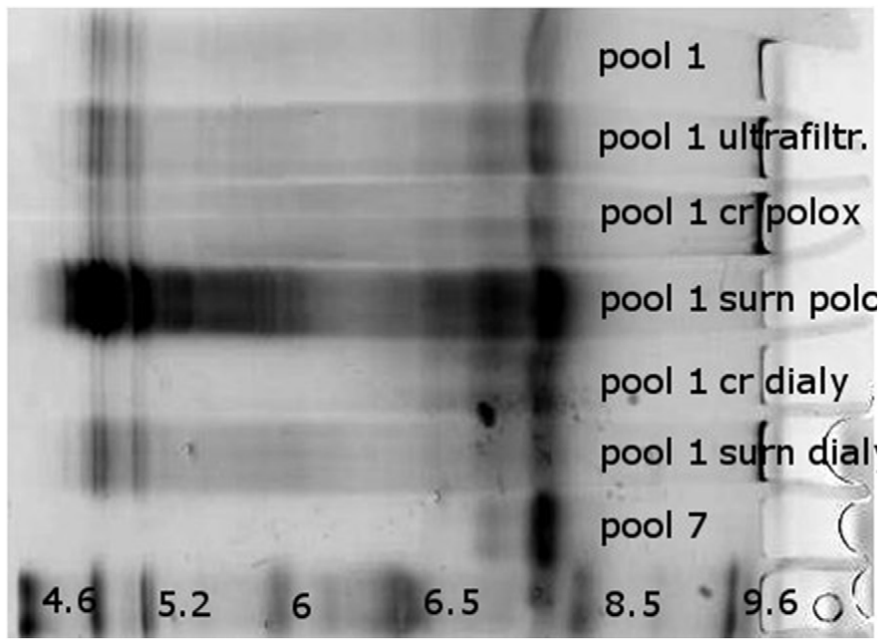
IEF analysis of the urate oxidase after crystallization. IEF analysis of the urate oxidase. From top to bottom: a) pool 1 ; b) pool 1 after 50 mM NH_4_Cl addition and ultrafiltration, c) crystal content from 35 mg/mL urate oxidase pool 1 with 5% poloxamer 188 in 5 mM Tris pH 8.5, 50 mM NH_4_Cl; d) supernatant from 35 mg/mL urate oxidase pool 1 with 5% poloxamer 188 in 5 mM Tris pH 8.5, 50 mM NH_4_Cl; d) pool 7 ; e) crystal content from 40 mg/mL urate oxidase pool 1 in 5 mM Tris pH 8.5, 50 mM NH_4_Cl, dialyzed against 5 mM Tris pH 8; f) supernatant from 40 mg/mL urate oxidase pool 1 in 5 mM Tris pH 8.5, NH_4_Cl 50 mM, dialyzed against 5 mM Tris pH 8, g) pool 7.

These first results suggest that, implementing a crystallization step in this purification process would have industrial advantages at two stages. It would need to be introduced after the first chromatography step (on pool 2 or 3), or preferably just before the first chromatography step (on pool 1). To test this hypothesis, we performed crystallization trials on both pool 1 and pool 3, this latter differing from pool 2 only by an ultrafiltration step and a change in the nature of the buffer. First, poloxamer 188, above its cmc, was added to urate oxidase pool 1. In pure solution of active urate oxidase (pool 7), 100 µm well-defined crystals have previously been obtained by a similar procedure [21]. Here, a solid form was obtained, but it appeared more like a precipitate than a crystal (Figure 4A). The precipitate was separated from the supernatant by centrifugation and the supernatant was pipetted. The precipitate was redissolved and SEC analysis (Figure 5A), while showing fewer impurities than in urate oxidase pool 1, indicates that the process is not as effective as the current chromatography step 1. In particular, aggregates cannot be removed. A decrease in proteins of low molecular weight is observed, but this is revealed to be due to the ultrafiltration step used to concentrate the solution prior to crystallization (data not shown). IEF analysis shows that the protein extracted by adding poloxamer presents the same IEF profile as pool 1, with slightly less protein having acidic isoelectric points (Figure 6). The activity test (Table 1) even shows only 25% of active protein retrievable, against 63% activity in pool 1, and 85% after the first chromatography step, which means that this procedure led to a loss of activity. No improvement was observed when PEG 8000 was used instead of poloxamer 188 in this procedure (data not shown).

**Table 1:** Efficacy of each technique in terms of % recovery.

	SEC (±2%)		IEF (±10%)	Activity (±5%)
	280 nm	226 nm		
Pool 1	51%	78%	45%	63%
Pool 2	85%	84%	65%	85%
From pool 1 crystals with poloxamer 188	67%	75%	47%	25%
From pool 1 crystals by reverse dialysis	90%	93%	70%	85%
Spontaneous pool 1 crystals	87%	96%	n.d.	n.d.
Pool 3	85%	84%	65%	85%
Pool 4	100%	100%	100%	100%
From pool 3 crystals with poloxamer 188	85%	84%	50%	98%
From pool 3 crystals by reverse dialysis	93%	92%	90%	100%

Comparison of the three techniques of purification, chromatography, crystallization by poloxamer addition, and crystallization by reverse dialysis in terms of % recovery of protein and activity.

n.d. not determined.

Crystallization trials were next performed using reverse salt dialysis (i.e. dialysis against the same buffer without salt) on urate oxidase pool 1. In pure urate oxidase solution (pool 7), well-defined 50 µm-sized crystals have been previously obtained in this way [22]. Here, 10 µm-sized solid forms were obtained. These are not as well shaped as pure crystals, but they no longer appear like precipitates (Figure 4B). These spherical protein particles (SPP) were separated from the supernatant by centrifugation and the supernatant was pipetted. The SPPs were redissolved and analysis of the solution, analyzed by SEC, showed fewer impurities than in pool 1, both for high molecular weight and low molecular weight molecules (Figure 5B). The overall purity of the urate oxidase obtained by reverse salt dialysis is comparable to what is obtained with the current step 1 of chromatography from a SEC perspective, even though the detailed profile differs in that it contains less high molecular weight molecules but more low molecular weight molecules. IEF confirms this result and shows that a fraction of the remaining low molecular weight proteins probably have acidic isoelectric points (Figure 6). The activity test shows 85% of active proteins –retrievable against 63% activity in pool 1 and 85% after the first chromatography step.

To investigate why adding polymer did not constitute an adequate purification process for urate oxidase, the same procedure was performed on pool 3, i.e. partially purified urate oxidase containing the active form (tetramer) and aggregates likely to be urate oxidase octamers. Pool 3 contains the same impurities as pool 2 and was chosen for crystallization experiments since, just like pool 1, it is in a buffer more suitable for crystallization trials (5 mM Tris pH 8.5, 0.1 mM EDTA). First, poloxamer 188 was added to urate oxidase pool 3. 10 µm-sized solid forms were obtained, not as well-shaped as crystals and appearing more like precipitates (Figure 4C). These solid forms were separated from the supernatant by centrifugation and the supernatant was pipetted. Solid forms were redissolved, and analysis of the solution by SEC, which showed that all impurities (octamers) are retained and that the process is ineffective (Figure 5C).

A second crystallization trial was performed using reverse salt dialysis of pool 3. 50 µm-sized well-shaped crystals were obtained (Figure 4D). These crystals were separated from the supernatant by centrifugation and the supernatant was pipetted. Crystals were redissolved, and the solution analyzed by SEC, which showed fewer impurities (octamers) than in pool 2 but still more than in pool 4 (Figure 5D). The overall purity of the urate oxidase obtained by reverse salt dialysis enables a decrease in octamer concentration, but is not as effective as the current second chromatography step.

These experiments on pool 3 confirm that adding polymer is less effective than reverse salt dialysis for purifying urate oxidase. Urate oxidase octamers and other type of aggregates seem to be as sensitive as urate oxidase to the depletion effect induced by polymer, thus explaining its lack of selectivity. Moreover, the strong salting-in effect exhibited by urate oxidase seems to be present to a lower extent in urate oxidase octamers, and absent in other types of aggregates and host cell proteins, which explains how purification can be achieved by this means.

**Figure 7:**
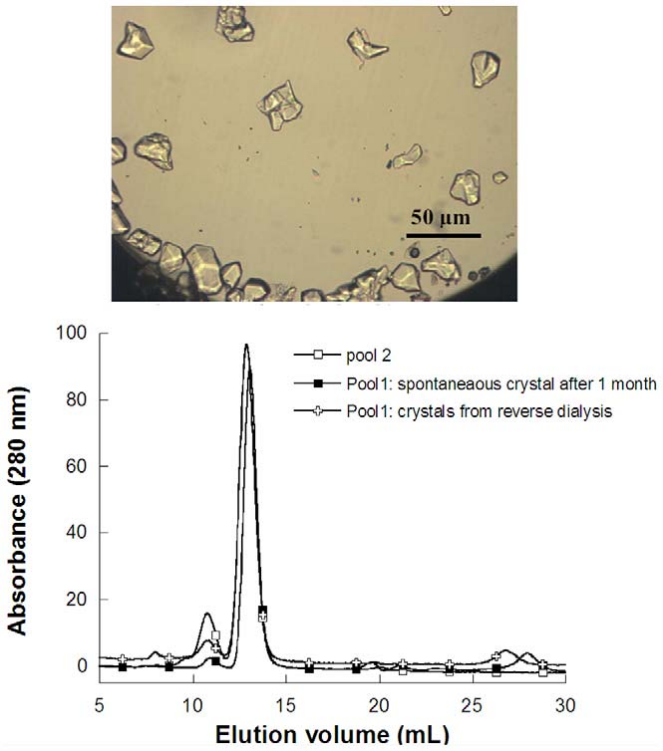
Spontaneaous urate oxidase crystals from pool 1 at 5°C (top) and SEC analysis (bottom). Top: Urate oxidase crystals grown spontaneously in pool 1 after one month's storage at 5°C. Bottom: SEC analysis of this urate oxidase crystal content grown in pool 1 after one month storage at 5°C (filled square) and comparison with crystals obtained after reverse dialysis from pool 1 (unfilled cross) and chromatography from pool 2 (unfilled square).

Overall, then, in the case of urate oxidase, purification based on salting-in crystallization is shown to be more effective than that by adding polymer. If the SEC results were confirmed by orthogonal analytical techniques, the purity of the protein obtained by crystallization in a low ionic strength buffer would challenge what is currently achieved with a first step of chromatography. This method can therefore be implemented during the current purification process with minimal process changes, since urate oxidase pool 1 is currently buffered at a low ionic strength (5 mM Tris pH 8.5; 0.1 mM EDTA) and the protein content (urate oxidase active form and impurities) is around 15 mg/mL. As the solubility of the active form of urate oxidase is 2 mg/mL (+/− 1 mg/mL) in these conditions, urate oxidase pool 1 is supersaturated and should eventually crystallize. In our experiment, when pool 1 was stored at 5°C, crystals were found to appear over time. Well-defined 20 µm-sized crystals were harvested after 1 month of storage (Figure 7, top) and a purity of 87%was assessed by SEC after redissolution of the crystals (Figure 7, bottom), which is comparable to what is obtained by the current first chromatography step. Further studies with a focus on nucleation kinetics are now needed to determine how this process can be improved (yield, crystal size distribution, robustness) scaled-up and accelerated: for instance, by seeding the solution with pure urate oxidase crystal fragments or by concentrating the solution to increase the nucleation rate.

### Summary

High-resolution crystallography techniques require crystallization of very pure macromolecules, and the present study confirms that crystallization is itself a relatively effective purification step, its efficacy depending primarily on the crystallization method used. Adding polymers to a solution of macromolecules can lead to its crystallization by depletion attraction between macromolecules. However this attractive interaction is more effective with large molecules, and adding polymer (poloxamer or polyethylene glycol) to urate oxidase solutions, either on the crude extract from fermentation broth or on the partially purified solutions, does not lead to satisfactory crystallization. Purification by this crystallization method is not as effective as a chromatography step, although it has been shown to be a very effective method for crystallization of pure protein solutions. This is probably due to the fact that large host cell proteins and urate oxidase aggregates are just as sensitive to the entropic depletion effect induced by high concentrations of poloxamer 188 and PEG 8000 as urate oxidase. The non-specific effect of polymers thus makes this crystallization method insufficiently selective for use as a purification step. In contrast, salts induce a more specific attraction on macromolecules in solutions [33]. Using salting-in and salting-out effects for purification by crystallization leads to very effective purification. Most proteins are sensitive to salting-out and this effect has been extensively studied [34]–[36] and used. The salting-in effect has only sporadically been used for crystallization [37], because this effect does not apply to all proteins [38]. Previous fundamental studies performed on pure urate oxidase and other proteins [22], [24], [39] revealed the nature of the interparticle forces which control the properties of macromolecule solutions and therefore their phase diagrams, and which ultimately generate crystals. Due to its strong salting-in effect, urate oxidase can be purified by crystallization from pool 1 as effectively as by current chromatography steps, at least from an SEC perspective.

This study therefore reveals that a crystallization approach to purification offers advantages in developing efficient industrial processes and reducing costs. Similar investigations on other readily available proteins of industrial interest should reveal the specific crystallization conditions relevant in each case.

## Materials and Methods

### Solutions

Solutions of recombinant urate oxidase (pool 1 to pool 7) were collected at different steps of the downstream process, which consists of four standard steps of chromatography (ion exchange, hydrophobic and gel filtration). In between these chromatography steps, ultrafiltration and diafiltration are also performed to concentrate the protein and exchange buffers. For reasons of confidentiality with regard to the current production process, details of columns and buffers cannot be disclosed.

1 M ammonium chloride and 0.015 mg/mL uric acid stock solutions were prepared by dilution of the appropriate quantity of the two salts (purchased from Sigma-Aldrich) in 50 mM Tris-HCl buffer, pH 8 (+/− 0.5 pH unit).

25% w/v = poloxamer 188 (from powder supplied by BASF) and 40% PEG 8000 (from 50% solution supplied by Hampton research) solutions were also prepared in 50 mM Tris buffer, pH 8.

All salt and urate oxidase solutions for crystallization trials were filtered through 0.22 µm Millipore filters.

### Size Exclusion Chromatography (SEC)

Size Exclusion Chromatography analyses were performed on a GE Healthcare AKTA basic system with a Superdex 200 GL column eluted in 50 mM sodium phosphate buffer pH 8. Seven proteins of molecular weights ranging from 13.7 kDa to 669 kDa were used to calibrate the column (ribonuclease, chymotrypsine, ovalbumine, aldolase, catalase, ferritine and thyroglobuline). Protein was detected by UV absorbance at 226 nm and 280 nm.

### Isoelectrofocusing (IEF)

All reagents were purchased from BIO-RAD: Criterion ready gel pH 5–8 and 3–10; 10 x anode buffer, 7 mM phosphoric acid; 10x cathode buffer 20 mM lysine, 20 mM arginine; sample buffer: 50% glycerol; gel stain Coomassie R-250/Crocein Scarlet and protein standard consisting of a mixture of nine native proteins with isoelectric points ranging from 4.45 to 9.6 (cytochrome c, lentil lectin, human hemoglobin C and A, equine myoglobin, human and bovine carbonic anhydrase, beta lactoglobulin B and phycocyanin). Twenty microliters of each sample (approximately at 1 mg/mL) were uploaded into the wells of the gel and power was applied (100 V constant for the first hour then 250 V constant for one hour and finally 500 V constant for 30 min). The gel was then bathed in the Coomassie staining solution for 45 min and washed overnight in the destaining solution (400 mL of 100% methanol, 500 mL of deionized water, +and 100 mL of glacial acetic acid).

### Biological activity test

The enzymatic activity of the urate oxidase was determined by monitoring the degradation of uric acid at 292 nm. Urate oxidase crystals grown either via salt dialysis or via polymer addition were dissolved in a solution of 50 mM Tris, pH 8, 100 mM KCl. The protein concentration was measured by UV absorbance at 280 nm, using an extinction coefficient of 1.69 mL.mg^−1^cm^−1^. The urate oxidase activity was determined by measuring the initial consumption rate of uric acid by spectrophotometry: 5×10^−5^ µg of urate oxidase was added to 6 µg of uric acid in 50 mM tris-HCl, pH 8.5, and the variation in concentration of the uric acid was monitered at 292 nm using an extinction coefficient of 12.2 mol^−1^ cm^−1^. The initial consumption rate, expressed in mol.min^−1^, was then normalized by the quantity of urate oxidase introduced (in mg) to obtain the specific activity of the enzyme. The experiment was repeated using two other concentrations of urate oxidase to correlate specific activity values.

### Crystallization trials

In Tris buffer, in the presence of 50 mM NH_4_Cl, pure urate oxidase is known [22] to be soluble to at least 100 mg/mL. NH_4_Cl was therefore added to impure urate oxidase solution from pools 1 or 3, to a final concentration of about 50 mM. These solutions were then concentrated to 50 mg/mL by ultrafiltration in a 10 mL Amicon cell using a 30 kDa cut-off membrane. Crystallization was performed either by adding poloxamer 188 to a final concentration of 2.5 to 4% or by dialyzing the solutions against 5 mM Tris pH 8 without salt using a 300 µL Spectra/Por Float-a-lyzer. In both cases, the solid phase (crystal or precipitate) obtained in batch after 12 h was separated from the supernatant by centrifugation and extraction of the supernatant by pipetting. The solid phase was redissolved in a 50 mM Tris pH 8, 100 mM KCl prior to analysis (SEC, IEF, activity test).
